# Edge Computing Driven Data Sensing Strategy in the Entire Crop Lifecycle for Smart Agriculture

**DOI:** 10.3390/s21227502

**Published:** 2021-11-11

**Authors:** Rihong Zhang, Xiaomin Li

**Affiliations:** College of Mechanical and Electrical Engineering, Zhongkai University of Agriculture and Engineering, Guangzhou 510225, China; zhangrihong@zhku.edu.cn

**Keywords:** data sensing, agricultural internet of things, data value, crop lifecycle, edge computing

## Abstract

In the context of smart agriculture, high-value data sensing in the entire crop lifecycle is fundamental for realizing crop cultivation control. However, the existing data sensing methods are deficient regarding the sensing data value, poor data correlation, and high data collection cost. The main problem for data sensing over the entire crop lifecycle is how to sense high-value data according to crop growth stage at a low cost. To solve this problem, a data sensing framework was developed by combining edge computing with the Internet of Things, and a novel data sensing strategy for the entire crop lifecycle is proposed in this paper. The proposed strategy includes four phases. In the first phase, the crop growth stage is divided by Gath-Geva (GG) fuzzy clustering, and the key growth parameters corresponding to the growth stage are extracted. In the second phase, based on the current crop growth information, a prediction method of the current crop growth stage is constructed by using a Tkagi-Sugneo (T-S) fuzzy neural network. In the third phase, based on Deng’s grey relational analysis method, the environmental sensing parameters of the corresponding crop growth stage are optimized. In the fourth phase, an adaptive sensing method of sensing nodes with effective sensing area constraints is established. Finally, based on the actual crop growth history data, the whole crop life cycle dataset is established to test the performance and prediction accuracy of the proposed method for crop growth stage division. Based on the historical data, the simulation data sensing environment is established. Then, the proposed algorithm is tested and compared with the traditional algorithms. The comparison results show that the proposed strategy can divide and predict a crop growth cycle with high accuracy. The proposed strategy can significantly reduce the sensing and data collection times and energy consumption and significantly improve the value of sensing data.

## 1. Introduction

In recent decades, in the background of the global population explosion, continuous urbanization, a sharp decrease in arable land area, and frequent extreme weather conditions, how to increase food production using limited land resources remains one of the most urgent and important issues in the agricultural field [1,2]. The Internet of Things (IoT), machine learning, artificial intelligence, unmanned aerial vehicles, and other new information technologies have been integrated into every part of modern agricultural production; the emergence and progress of new modern agricultural paradigms, such as smart agriculture, and unmanned farm have been greatly promoted [3,4,5,6,7]. The new agricultural paradigm centers on all data types during the entire lifecycle of crops and realizes detailed and intelligent control of agriculture. Therefore, how to complete fast and efficient sensing of crop and environmental parameters in different growth stages of crops has become an important basis for promoting the implementation and rapid progress of agricultural models.

After extensive academic and industry research, different frameworks and methods for crop data sensing have been proposed. In general, the following stages have been experienced. At first, the monitoring of related parameters of agricultural crops was mainly based on manual measurement of field parameters, such as soil temperature, humidity, and crop leaf area. However, this approach has the disadvantages of high labor intensity and low automation level [8]. With the introduction of sensor technology, wireless communication, and other technologies, this approach has entered the wireless-sensing stage based on static IoT. In other words, by deploying wireless sensing nodes with a small number of sensors in crop planting areas, agricultural IoT has been introduced to complete the crop parameters sensing [9]. However, the mentioned scheme still has a static monitoring system and a lack of mobility. To overcome these shortcomings, researchers have upgraded sensing nodes on the basis of the traditional IoT and introduced unmanned aerial vehicle (UAV) and multisensor systems. Agricultural data sensing has ushered in the current stage of multiparameter and mobile sensing [10,11].

Although agricultural wireless sensor networks (WSNs) have made significant progress in the field of agricultural data sensing, there has been little relevant research on entire crop lifecycle data sensing. At present, the field of agricultural data sensing faces the following challenges. The first challenge is the limited working time of the data sensing system due to limited energy. Despite advances in battery technology, the number of nodes used in the entire lifecycle of crops has significantly increased the types and size of sensing data. Sensing nodes have to deal with many tasks, but these nodes have relatively limited working time. The second challenge relates to the low density and relevance of the collected data. Although many parameters can be collected by a sensing network, different growth stages require different sensing data types. The existing methods do not consider the relevance between sensing data types and growth stages, hence, the collected data have low density and relevance. The third challenge relates to ignoring the overlapping area of sensing nodes. In traditional methods, a large number of sensing nodes are used. A large number of nodes means overlapping of sensing areas and redundancy of sensing data and, therefore, a decrease in the value of sensing data [12,13]. As a result, the sensing network has to spend more energy on collecting low-value data. The fourth challenge relates to the lack of intelligence of the current strategies. As the sensing nodes have low computing and storage capabilities, they can neither analyze sensing data nor adjust and optimize the sensing data type in realtime. Thus, it is urgent to develop new sensing frameworks and methods.

The abovementioned challenges of crop lifecycle data sensing of the traditional agricultural IoT require new solutions. To this end, a new crop parameter sensing framework was constructed by integrating edge computing and IoT, and an entire crop lifecycle data sensing strategy is proposed.

This study has three specific contributions to new knowledge about crop modeling. First, a lightweight entire crop lifecycle data sensing framework is proposed based on IoT and edge computing, and the data driven algorithms are used in the edge server to optimize sensing parameters and increase data values by reducing redundancy and enhancing the correlations between sensing data and crop growth stages; these algorithms can be found in Section 4.2–Section 4.4. Second, the method of crop growth stage division and key feature selection and the crop growth stage prediction model based on a neural network are proposed. Third, an adaptive sensing method of sensing nodes is established.

Section 2 reviews related work on agricultural data sensing and edge computing. Section 3 introduces the new data sensing framework and working principle. Section 4 introduces the mathematical model and data sensing strategy. Section 5 presents the experimental results. Section 6 concludes the paper.

## 2. Related Work

### 2.1. Application of Edge Computing in Agriculture

With the advantages of being close to the equipment, good realtime performance, and strong computing capability, edge computing as a new computing paradigm has been highly used by academics, enterprises and in the agricultural field [14,15,16,17]. Caria et al. [18] constructed an intelligent pasture monitoring system for monitoring livestock and pasture environment based on edge computing, where an edge computing unit was designed using the raspberry PI as a computing module. Ferrández-Pastor et al. [19] designed a precision agriculture distributed computing framework based on the IoT by integrating edge computing and other new information technologies and conducted practical implementation and relevant experiments in greenhouses. Bu et al. [20] proposed an agricultural IoT framework integrating edge computing. The edge computing layer was used to monitor the working state of the sensors at the physical layer and compress sensing data. Debauche et al. [21] designed a platform for edge computing for monitoring a poultry breeding environment based on an Nvidia Jetson Nano microcomputing unit. In the abovementioned literature on edge computing, the location of computing resources is fixed, which makes it difficult to meet the needs of large-scale agricultural production. To overcome this limitation, mobile edge computing has been introduced [22,23]. Uddin designed an UAV mobile edge computing platform to meet the needs of large-scale farming [24]. However, the agricultural IoT frameworks based on edge computing and related primary applications are still in the initial stages  [25]. There have been few related studies on improving the parameter sensing performance and data value in the entire crop lifecycle using edge computing [26].

### 2.2. Data Sensing for Smart Agriculture

Agricultural data sensing is fundamental for optimizing field management and realizing precise agricultural control. Agricultural data collection has developed from manual field measuring to wireless automatic sensing, of which agricultural WSNs and agricultural IoTs are typical representatives [27,28]. Pallavi et al. [29] proposed a parameter remote sensing system based on agricultural IoT for greenhouses and realized intelligent opening and closing control of greenhouse windows considering sensing data. Happila et al. [30] built a remote crop monitoring platform based on a traditional agricultural sensor network by integrating cameras to monitor crops and their growth environment in real time. Cubero et al. [31] constructed a remote-controlled field robot equipped with color, multispectral, and hyperspectral cameras to detect diseases and insect pests on horticultural crops by using proximal sensing technology. Popescu et al. [32] proposed a hierarchical system based on the combination of UAV and WSNs for crop monitoring and parameter collection in precision agriculture. Marchese et al. [33] introduced the idea of integrating UAV, 5G, and an artificial satellite to construct a three-dimensional network for agricultural information collection. Munir et al. [34] introduced edge computing nodes between traditional agricultural IoT sensing nodes and remote servers to optimize irrigation sensing data. Furthermore, in [26], the authors proposed a new data sensing model based on high-performance edge computing to realize efficient unloading of data and smooth workflow optimization for monitoring crop soil environment.

The abovementioned studies have provided a necessary reference for agricultural parameter sensing, but there have been few related studies on the entire growth cycle of crops and parameter sensing optimization.

## 3. Agricultural Data Sensing Framework

### 3.1. Data Sensing Framework Structure

**Figure 1:**
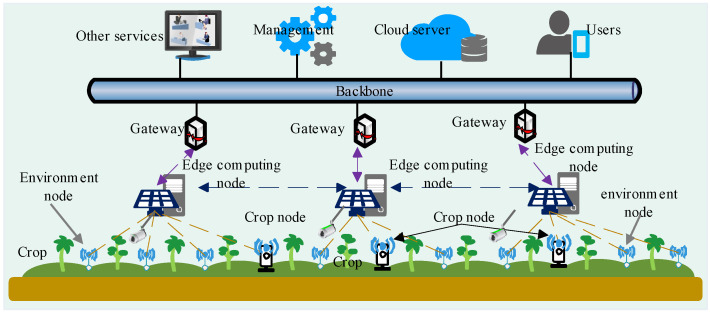
The data sensing framework for the entire crop lifecycle based on IoT and edge computing.

Combining the advantages of traditional IoT, sensors, and edge computing, a smart agricultural parameter sensing system was proposed, as shown in Figure 1. The proposed framework includes four layers: a sensing layer, an edge computing layer, a network layer, and an application layer. Specifically, the sensing layer is composed of multiple sensing nodes, which are equipped with a variety of sensors, and power, memory, microprocessing, and wireless communication modules. According to node functions, the sensing nodes in the proposed framework can be divided into crop monitoring nodes and environmental sensing nodes, which are used to sense crop growth and environmental parameters, respectively. The edge computing layer consists of multiple edge computing nodes, and to improve the working time of these nodes, they were equipped with rechargeable units. The network layer includes wired and wireless media, different communication protocols, and a variety of gateway, routing, and other equipment. This layer is responsible for connecting the sensing layer, edge computing layer, and application layer, and it provides communication between different layers and devices. The application layer is centered on the cloud server, including agricultural users and terminals, intelligent monitoring and control, and other applications and services. In particular, cluster topology is used to complete the networking in the sensing layer, and each cluster takes an edge computing node as a cluster head that manages the network resources of the cluster.

### 3.2. Working Principle

The proposed agricultural data sensing framework operation was divided into the following steps. First, based on the historical data, the cloud server established a crop expert system and classification standards for different stages of the entire lifecycle of crops, evaluated the crop growth indicators corresponding to each growth stage, and sorted the growth indicators according to their priority. At the same time, the environmental parameters under the main growth index of crops at different stages were analyzed regarding the correlation degree, and the environmental parameters with a high correlation degree at different growth stages were extracted as priority sensing environmental parameters. Second, an edge computing node obtained working parameters of the expert system and different nodes from the cloud server, drove the crop monitoring node to collect the current crop-related parameters periodically, such as sensing leaf area or stem height in different crop growth stages, at the preset frequencies, and collected the sensing data from different nodes. The edge computing node used an artificial neural network learning algorithm to identify the current growth stage and queried the key growth indicators of crops corresponding to the stage. The correlation degrees between the environmental parameters and growth indicators were calculated and the environmental parameters were sorted according to the correlation degrees. Third, the edge computing node selected the key feature parameters of crops and environmental parameters that met the sensing time constraint and selected the sensing node set with coverage and the number of nodes participating as the condition. Finally, the sensing nodes were driven to complete parameter sensing and upload the data to the edge computing node and cloud server.

## 4. System Model and Method

### 4.1. System Model

Based on the framework shown in Figure 1, the system model is introduced. In the monitoring region with a notation Λ, let $Ar_{\Lambda }$ be the area of Λ and assume there were many sensor nodes deployed in Λ. The sensor nodes were organized into clusters. Let $C=\{c_{1},c_{2},c_{3},\cdots \}$ be the sensing cluster set. Each cluster has an edge computing node and many sensor nodes, and each node can sense multiple parameters. All edge computing nodes have the same performance, structure, function, and performance. Let $L=\{l_{1},l_{2},l_{3},\cdots \}$ be the growth stage set. For any cluster c∈C, in any growth stage l∈L, the sensing data type set can be divided into the crop growth index set and environmental parameter set $Z=\{z_{1},z_{2},z_{3},\cdots \}$.

Assume that the value vector of the environmental parameters is expressed as $V\left(z\right)=\left(v_{1},v_{2},v_{3},\cdots \right)$; then, it is easy to obtain the total data value of environmental parameters as follows:

(1) $$V\left(Z\right)=\sum_{j\in f}v(z_{j}),$$

where $v(z_{j})$ denotes the *j*th environmental parameter value.

**Definition** **1.** *Effective sensing area $\left(Ar_{v}\right)$ represents the difference between the total sensing area $\left(Ar_{M}\right)$ and overlapping sensing areas $\left(Ar_{over}\right)$, which can be expressed as follows:*

(2) $$Ar_{v}=Ar_{M}-Ar_{over}.$$

According to Definition 1, the effective value of the sensing environment data (V(c,Z)) can be obtained as:

(3) $$V\left(c,Z\right)=\frac{Ar_{v}}{Ar_{\Lambda }}V\left(Z\right).$$

Without loss of generality, for a cluster *c* in a growth stage *l* , the numbers of sensing nodes of the crop growth index and environmental parameters are denoted by *g* and *q*, and the numbers of the corresponding sensing parameters are denoted by *h* and *f*, respectively. Therefore, the sensing times of crop index (t(X)) and environmental parameters t(Z) can be, respectively, calculated by:

(4) $$t\left(X\right)=\sum_{i\in h}t(x_{i}),$$

(5) $$t\left(Z\right)=\sum_{j\in f}t(z_{j}),$$

where $t(x_{i})$ and $t(z_{j})$ represent the sensing time of the *i*th crop growth index and the *j*th environmental parameter, respectively.

Assume that the communication rate is $v_{c}$ , then the total data collection times of crop growth indexes and environmental parameters can be, respectively, computed as:

(6) $$t_{c}\left(x\right)=g\cdot \sum_{i\in h}\frac{ds(x_{i})}{v_{c}}+t\left(X\right),$$

(7) $$t_{c}\left(Z\right)=q\cdot \sum_{j\in f}\frac{ds(z_{j})}{v_{c}}+t\left(Z\right),$$

where $ds(x_{i})$ and $ds(z_{j})$ represent the data sizes of the *i*th crop growth index and the *j*th environmental parameter, respectively.

Then, the main problem can be defined as how to sense data with the highest total value at a given time. Furthermore, according to the sensing data type, this problem can be decomposed into how to obtain the crop growth index data and environmental parameter data with the maximum value under the time constraint.

### 4.2. Division of Crop Growth Stages and Selection of Key Features Based on GG Fuzzy Clustering

#### 4.2.1. Division of Crop Growth Stages

To realize fine division of the crop growth cycle and to improve the information value of sensing data, a method of crop growth stage division was developed based on GG fuzzy clustering [35]. It was assumed that the collected crop growth index data can be divided into *n* time slots; then, the historical data set of its time series can be defined as $XL=\{XL_{1},XL_{2},\cdots ,XL_{n}\}$. Furthermore, the growth stage can be divided into *k*(2≤k≤n) stages. The $XL_{j}\in XL$ is the *j*th slot crop growth index vector with *h* growth parameters. The partition matrix is given as $U={\left[u_{ij}\right]}_{k\times n}$ , where i=1,2,⋯,k; j=1,2,⋯,n, and $u_{ij}\in \left[0,1\right]$ is the membership degree of the *j*th time slot crop growth index belonging to the *i*th growth stage. The cluster center set is expressed as $CO=\{co_{1},co_{2},\cdots ,co_{k}\}$. In addition, a classification coefficient and average fuzzy entropy were used to evaluate the clustering effect.

**Definition** **2.** *Classification coefficient (CC) is defined as the square mean value of membership degree and is calculated by (8). The closer the classification coefficient is to one, the better the clustering effect is.*

(8) $$\alpha =\frac{1}{n}\sum_{i\in k}\sum_{j\in n}u_{ij}$$

**Definition** **3.** *Average fuzzy entropy (AFE) is defined as an information entropy contained in the membership degree distribution, and it is calculated by (9). The closer the average fuzzy entropy is to zero, the better the clustering effect is.*

(9) $$\beta =-\frac{1}{n}\sum_{i\in k}\sum_{j\in n}u_{ij}\ln u_{ij}$$

Based on the above assumptions and definitions, the division algorithm of crop growth stages based on GG fuzzy clustering included the following steps:

- Step 1: Set the terminal parameter ε(ε>0); initialize randomly the partition matrix $U_{k\times n}^{0}$; set the cluster number *k* = 2, the number of iterations ς(ς=1,2,3,⋯), and fuzzy weighting parameter m∈(1,+∞).
- Step 2: Calculate the clustering center by:
  (10) $$co_{j}^{\varsigma }=\sum_{i=1}^{n}{\left({u_{ij}}^{\left(\varsigma -1\right)}\right)}^{m}XL_{i}/\sum_{i=1}^{n}{\left({u_{ij}}^{\left(\varsigma -1\right)}\right)}^{m}.$$
- Step 3: Calculate the covariance matrix and prior probability of each cluster, respectively, by:
  (11) $$B_{j}^{\left(\varsigma \right)}=\sum_{i=1}^{n}u_{ij}^{\left(\varsigma -1\right)}\left(XL_{i}-co_{j}^{\left(t\right)}\right){\left(XL_{i}-co_{j}^{\left(t\right)}\right)}^{T}/\sum_{i=1}^{n}u_{ij}^{\left(\varsigma -1\right)};1\leq j\leq k,$$
  (12) $$P_{j}^{\varsigma }=\sum_{i=1}^{n}({u_{ij}}^{\left(\varsigma -1\right)})/n.$$
- Step 4: Calculate the fuzzy maximum likelihood distance by:
  (13) $$D{\left(XL_{i},co_{j}\right)}_{B_{j}}=\sqrt{\det(B_{j})}\exp\left[{\left(XL_{i}-co_{j}^{\varsigma }\right)}^{T}B_{j}^{-1}\left(XL_{i}-co_{j}^{\varsigma }\right)/2\right]/p_{j}.$$
- Step 5: Update the matrix by:
  (14) $$\;u_{ij}^{\varsigma }=1/\sum_{\eta =1}^{k}{\left(D{\left(XL_{i},c_{j}\right)}_{B_{j}}/D{\left(XL_{i},c_{\eta }\right)}_{B_{j}}\right)}^{2/(m-1)}.$$
- Step 6: Repeat Steps 2–5; update the partition matrix, and when $|U^{\varsigma }-U^{\varsigma -1}\left|\right|<\varepsilon$, stop updating. Then, calculate CC and AFE.
- Step 7: Increase *k* by one, *k* = *k*++; repeat Steps 2–6, and when *k* = *n* , stop repeating. Search for the division results corresponding to optimal CC and AFE.
- Step 8: According to the optimal clustering results, calculate the corresponding growth stage division of crops $L=\{l_{1},l_{2},l_{3},\cdots ,l_{k}\}$ and growth index time series set.

To this point, the growth stage of the entire crop lifecycle can be obtained by the method based on historical data.

#### 4.2.2. Key Features Selection

Without loss of generality, $XL\left(\lambda ,l\right)=\left\{xl_{\lambda 1},xl_{\lambda 2},\cdots ,xl_{\lambda \eta }\right\}$ is the time series data of the λth(λ∈h) standardized crop growth index with η(1≤η≤n) time slots. Variance is used to represent the change in the growth index vector in the growth stage; the greater the variance is, the greater the change in the crop growth index in that stage will be. Therefore, the variance of XL(λ,l) can be obtained by:

(15) $$\sigma^{2}\left(\lambda ,l\right)=\frac{\sum {\left(xl_{\lambda i}-\mu \left(\lambda ,l\right)\right)}^{2}}{\eta },$$

where $xl_{\lambda i}\in XL\left(\lambda ,l\right)\;\left(1\leq i\leq \eta \right)$ is the *i*th variable and μ(λ,l) is the mean value. Repeat the above steps to obtain the variance vector $\Delta =\{\sigma_{1}^{2},\sigma_{2}^{2},\cdots ,\sigma_{h}^{2}\}$ of *h* crop growth indicators in stage *l* of crop growth. Then, the crop indexes that meet the condition (16) are selected as key features corresponding to growth stage *l*.

(16) $$\sigma_{j}^{2}>\theta \;\left(\sigma_{j}^{2}\in \Delta \right)$$

In (16), θdenotes the variance boundary condition.

Finally, sort the key features in order from the largest to the smallest and obtain the key features vector with the priorities decreasing in sequence $\bar{X(l)}=\left\{\bar{x_{1}},\;\bar{x_{2}},\;\bar{x_{3}},\cdots \right\}$.

### 4.3. Crop Growth Stage Prediction Based on a Neural Network

This section considers the specific growth stage prediction using the current sensing data and selecting the crop growth sensing parameter with a time constraint. First, all crop parameters were collected. Then, the growth stage prediction was performed using a neural network. Finally, the crop growth sensing parameter with the time constraint was selected.

A T-S fuzzy neural network (T-S FNN) [36] was adopted for predicting the growth stage. The T-S FNN network uses the “if-then” rule, which is defined as follows:

(17) $$\begin{array}{c} R^{i}:\mathrm{if}\;x_{1}\;\mathrm{is}\;A_{1}^{i},x_{2}\;\mathrm{is}\;A_{2}^{i},x_{3}\;\mathrm{is}\;A_{3}^{i}\cdots \;\mathrm{then}\\ y_{i}=p_{0}^{i}+p_{1}^{i}x_{1}+p_{2}^{i}x_{2}+p_{3}^{i}x_{3}+\cdots , \end{array}$$

where $A_{j}^{i}$ denotes the fuzzy set, $p_{j}^{i}\left(j=1,2,\cdots ,h\right)$ is a set of fuzzy parameters, and $y_{i}$ the fuzzy output.

Antecedent network fuzzy layer design: Let $x=[x_{1},x_{2},\cdots ,x_{h}]$ be the sensing data for all indicators of collected crops. The membership degree of input $x_{j}$ is calculated using the Gaussian function as follows:

(18) $$\mu_{A_{j}^{i}}=\exp\left(-{\left(x_{j}-o_{j}^{i}\right)}^{2}/b_{j}^{i}\right)\;\left(1\leq j\leq h;\;1\leq i\leq N\right),$$

where $o_{j}^{i}$ and $b_{j}^{i}$ denote the center and width of the membership function, respectively; *N* is the number of fuzzy subsets.

The fuzzy calculation is performed for each membership degree, and the applicability of each fuzzy rule is obtained by using fuzzy operator as a multiplication operator, as follows:

(19) $$\omega^{i}=\prod_{j=1}^{h}\mu_{A_{j}^{i}}\left(x_{j}\right).$$

The normalized output value $y_{i}$ of the fuzzy model is calculated according to the fuzzy calculation results by:

(20) $$y_{i}=\sum_{i=1}^{N}\omega^{i}\left(p_{0}^{i}+p_{1}^{i}x_{1}+\cdots +p_{h}^{i}x_{h}\right).$$

Posterior network parameter adjustment: The learning process of the T-S FNN is as follows.

(1) Error calculation: $y_{d}$ and $y_{T-S}$ are the expected and real outputs of the T-S FNN, respectively. The error *e* can be calculated by:

(21) $$e=\frac{1}{2}{\left(y_{d}-y_{T-S}\right)}^{2}.$$

(2) FNN parameters update: The FNN parameters are updated by:

(22) $$p_{j}^{i}\left(\vartheta \right)=p_{j}^{i}\left(\vartheta -1\right)-\ell \frac{\partial e}{\partial p_{j}^{i}},$$

(23) $$\frac{\partial e}{\partial p_{j}^{i}}=\frac{\left(y_{d}-y_{c}\right)\omega^{i}}{\sum_{i=1}^{N}\omega^{i}x_{j}},$$

where $p_{j}^{i}$ denotes the FNN parameters, *ℓ* is the learning rate, and ϑ is the training time.

(3) Modification of membership degree function parameter: Let $o_{j}^{i}$ and $b_{j}^{i}$ be the center and width of the membership degree function, respectively; γ is the learning momentum coefficient. The modification of the membership degree function parameter can be defined as:

(24) $$o_{j}^{i}\left(\vartheta \right)=o_{j}^{i}\left(\vartheta -1\right)-\gamma \frac{\partial e}{\partial o_{j}^{i}},$$

(25) $$b_{j}^{i}\left(\vartheta \right)=b_{j}^{i}\left(\vartheta -1\right)-\gamma \frac{\partial e}{\partial b_{j}^{i}}\cdot$$

**Figure 2:**
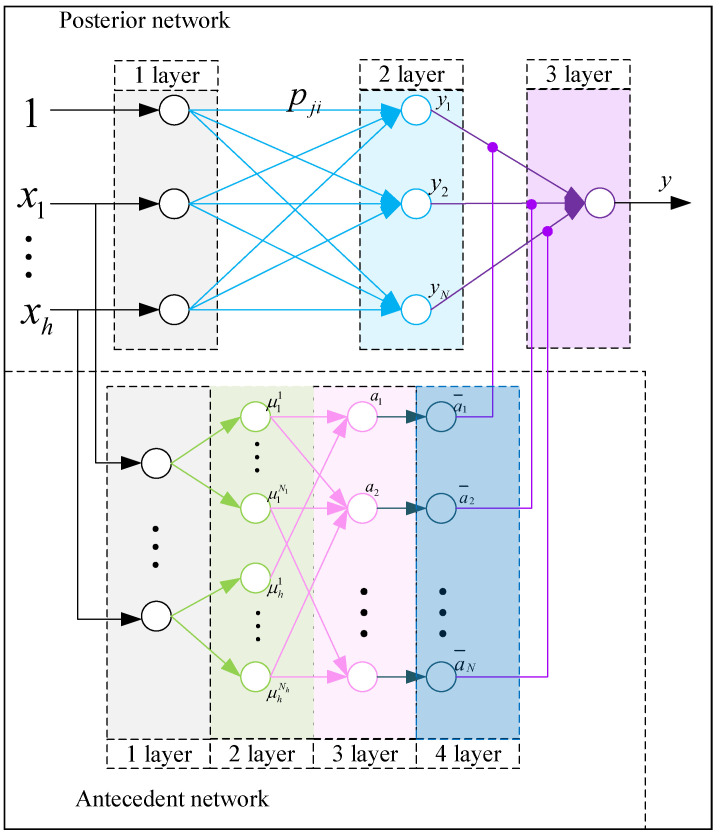
The T-S FNN structure for predicting the crop growth stage.

The structure of a fuzzy neural network used for predicting crop growth stage is shown in Figure 2. After training and testing, a stable T-S fuzzy neural network was obtained. The obtained network was consistent with the actual crop growth stage division and evaluation, and it was downloaded to each edge computing node.

The edge computing node uses the T-S FNN to predict the growth stage and to obtain the current growth stage l∈L and the corresponding growth parameters $\bar{X(l)}=\left\{\bar{x_{1}},\;\bar{x_{2}},\;\bar{x_{3}},\cdots \right\}$. Then, the sensing parameters $\dot{X}\left(l\right)=\left\{{\dot{x}}_{1},{\dot{x}}_{2},{\dot{x}}_{3},\cdots \right\}$ of each node with the time constraint are obtained by:

(26) $$\dot{X}\left(l\right)=\left\{{\bar{x}}_{i}|\sum_{i\in h}t({\bar{x}}_{i})\leq t_{\lim}\left(X,l\right)\right\},$$

where $t_{\lim}\left(X,l\right)$ is the time constraint for sensing the growth index in the growth stage *l*.

### 4.4. Environmental Sensing Parameter Optimization Based on Deng’s Grey Correlation Analysis

Since there are many environmental parameters of crop growth, direct selection of key environmental parameters affects the sensing data value. Therefore, based on the grey correlation analysis [37], the correlation degree between different growth indicators and environmental factors was calculated to obtain the environmental factors with the greatest influence.

Further, $X_{0}=\left\{x_{0}\left(\tau \right),\tau =1,2,\cdots ,n\right\}$ is the crop growth index data of *n* time slots, and it represents the reference sequence; $Z_{i}=\left\{z_{i}\left(\tau \right),\tau =1,2,\cdots ,n\right\}$ is the environment data of *n* time slots, and it represents the comparison sequence. Therefore, the grey correlation coefficient ξ between $x_{0}\left(\tau \right)$ and $z_{i}\left(\tau \right)$ can be calculated by:

(27) $$\xi \left(z_{0}\left(\tau \right),x_{i}\left(\tau \right)\right)=\frac{\min_{i}\min_{i}|x_{0}\left(\tau \right)-z_{i}\left(\tau \right)|+\rho \max_{i}\max_{i}\left|x_{0}\left(\tau \right)-z_{i}\left(\tau \right)\right|}{|x_{0}\left(\tau \right)-z_{i}\left(\tau \right)|+\rho \max_{i}\max_{i}\left|x_{0}\left(\tau \right)-z_{i}\left(\tau \right)\right|},\;$$

where ρ∈[0,1] is the resolution parameter. The degree of grey correlation (DGC) between $X_{0}$ and $Z_{i}$ is obtained as:

(28) $$\zeta \left(X_{0},Z_{i}\right)=\frac{1}{n}\sum_{\tau =1}^{n}\xi (x_{0}\left(\tau \right),z_{i}\left(\tau \right)).\;$$

The closer the DGC value is to one, the better the correlation between the environmental parameter and crop growth index will be. However, the abovementioned methods consider only the correlation between a certain index and environmental parameters. In practical applications, the priority of crop indicators must be considered. For this reason, the weight of the crop growth index was considered to optimize the grey correlation degree; ${\dot{X}}_{j}$ is the growth index set of stage *l*; the growth index weight degree of grey correlation (GIW-DGC) between ${\dot{X}}_{j}$ and $Z_{i}$ can be obtained as:

(29) $$\zeta \left({\dot{X}}_{j},Z_{i}\right)=\frac{\sigma^{2}\left({\dot{X}}_{j}\right)}{\sum_{j=1}^{|\dot{X}\left(l\right)|}\sigma^{2}\left(\dot{X}\right)}\cdot \frac{1}{n}\sum_{\tau =1}^{n}\xi (x_{j}\left(\tau \right),z_{i}\left(\tau \right))\cdot$$

The edge computing nodes repeat the above-presented steps to obtain the GIW-DGC between $\bar{X(l)}=\left\{\bar{x_{1}},\;\bar{x_{2}},\;\bar{x_{3}},\cdots \right\}$ and all environmental parameters. Then, the GIW-DGC is ranked, and the environmental parameters to sense are selected. The details of the method are given in Algorithm 1.
**Algorithm 1:** Environmental parameter selection based on grey correlation analysis
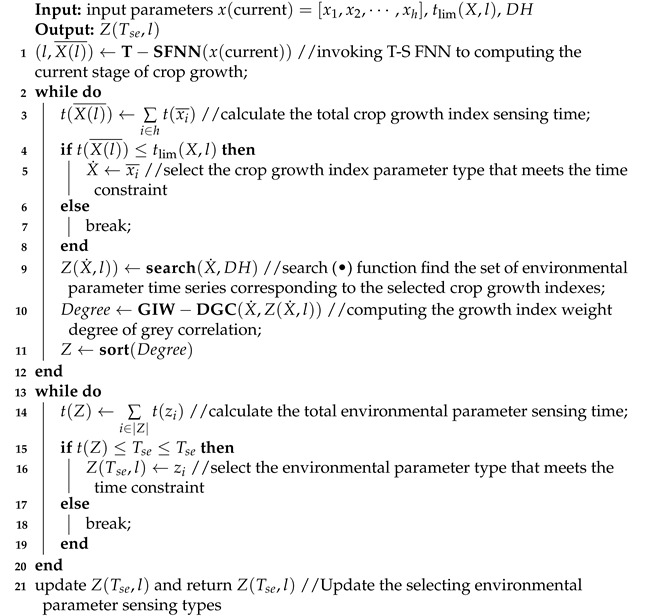


First, edge computing nodes drove the sensor nodes to sense *h* crops’ growth index $x\left(\mathrm{current}\right)=\left[x_{1},x_{2},\cdots ,x_{h}\right]$. Then, edge computing used the T-S FNN model to predict the growth stage *l* and the corresponding key growth feature $\bar{X(l)}$. Second, the sensing parameter type $\dot{X}$ was selected from while meeting the constraint ($t\left(\bar{X(l)}\right)\leq t_{\lim}\left(X,l\right)$). Third, we selected $Z(\dot{X},l)$ from the historical data set DH. Next, we computed and sorted the GIW-DGC between $\dot{X}$ and $Z(\dot{X},l)$. Then, we selected the greatest GIW-DGC as the sensing environmental parameters successively until meeting the time constraint $T_{se}$. Finally, the parameters were updated, and the results were returned.

### 4.5. Adaptive Sensing with Effective Sensing Area Constraint

In the previous section, the set of crop growth sensing parameter type $\dot{X}$ and the sensing time environmental parameters $Z(T_{se},l)$ of a growth stage *l* were obtained. Next, the adaptive sensing with effective sensing area constraint is introduced for sensing nodes. Let $S\left(c\right)=\left\{s_{1},s_{2},s_{3},\cdots \right\}$ be the sensor set in a sensor node cluster *c*, the set of node sensing times N={n1,n2,n3,⋯}, and the location set P={(x1,y1),(x2,y2),⋯}. For a sensor node s∈S(c), its location is (x,y) and its sensing time is n.

Assume that the set of selected sensing nodes set is $\dot{S}=\left\{{\dot{s}}_{1},{\dot{s}}_{2},{\dot{s}}_{3},\cdots \right\}$, and the set of candidate sensing nodes is $S_{\mathrm{left}}=S\left(c\right)-\dot{S}$ . Then, the centroid (x˙,y˙) coordinates of $\dot{S}$ can be obtained as follows:

(30) x˙=∑i∈|S˙|xi|S˙|,y˙=∑i∈|S˙|yi|S˙|.

The distance between $s_{\mathrm{left}}\left(s_{\mathrm{left}}\in S_{\mathrm{left}}\right)$ and (x˙,y˙) can be obtained by:

(31) d(sleft,S˙)=(xleft−x˙)2+(yleft−y˙)2,

where $s_{\mathrm{left}}$ is the candidate sensor node and (xleft,yleft) indicates its location.

By repeating (31), the distance set $D(S_{\mathrm{left}},\dot{S})$ between all members of $S_{\mathrm{left}}$ and the centroid can be obtained, and the set of sensing times of the candidate sensor node N(Sleft) can be obtained. Further, we can denote the normalized results of $D(S_{\mathrm{left}},\dot{S})$ and N(Sleft), respectively. The score $gd(s_{\mathrm{left}})$ of $s_{\mathrm{left}}$ can be obtained by:

(32) gd(sleft)=d¯(sleft,S˙)d¯max+n¯minn¯(sleft),

where n¯min is the minimum value of N¯(Sleft).

Repeating the above steps, the maximum value score $gd_{\max}$ and the corresponding sensor node $s_{\mathrm{left}}\left(gd_{\max}\right)$ can be obtained. Then, the related sets are updated by $S_{\mathrm{left}}=S_{\mathrm{left}}-s_{\mathrm{left}}\left(gd_{\max}\right)$ and $\dot{S}=\dot{S}+s_{\mathrm{left}}\left(gd_{\max}\right)$. The above steps are included in the *nodeSearch*(•) function. According to (2), an effective sensing area $Ar_{v}\left(\dot{S}\right)$ of $\dot{S}$ can be obtained. The updating process of $\dot{S}$ stops when the following condition is met:

(33) $$Ar_{v}\left(\dot{S}\right)\geq Ar_{\lim},$$

where $Ar_{\lim}$ is the minimum effective sensing area in a particular application. According to the aforementioned parameters and steps, the method that is shown in Algorithm 2 can be constructed to complete adaptive sensing for the sensor nodes.

The specific steps are as follows. First, input the set and parameters S(c), N, P, *l*, $Ar_{\lim}$, and then initialize $\dot{S}$, $S_{\mathrm{left}}$ , and other parameters. Second, choose the first sensor node corresponding to the minimum sensing time to join the selected node set $\dot{S}$. Third, invoke the *nodeSearch*(•) function to return $s_{\mathrm{left}}\left(gd_{\max}\right)$ to join $\dot{S}$. Meanwhile, calculate $Ar_{v}\left(\dot{S}\right)$ and determine whether it meets the constraint. Fourth, repeat Step 3 until $Ar_{v}\left(\dot{S}\right)\geq Ar_{\lim}$. Then, update N and $\dot{S}$ . Finally, the edge computing nodes establish wireless connections with selected sensing nodes and drive the selected nodes’ complete parameter N sensing and upload to the edge server. The proposed method is applicable to both environmental parameters and crop growth index parameters.
**Algorithm 2:**An adaptive working strategy of sensing nodes with effective sensing area constraints
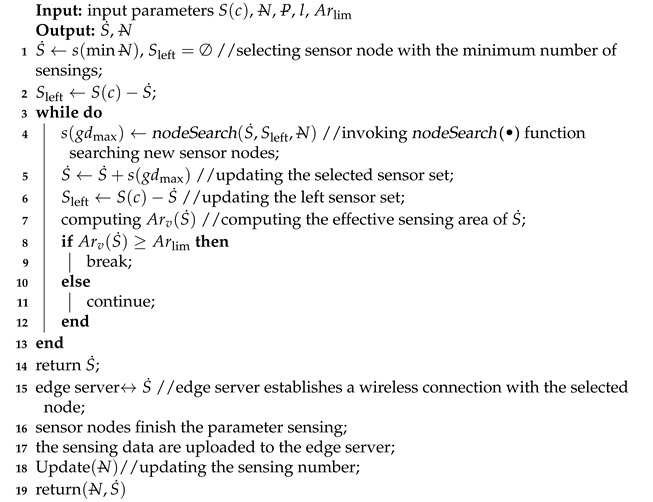


## 5. Experimental Results

### 5.1. Experimental Setup and Parameters

In the experiment, the growth indexes and environmental parameters of lettuce in the whole growth cycle were considered, and 1500 historical datasets were established. The data were randomly divided into three groups of data at different growth stages (I(20); II(30); III(20)) and used as test data. Based on the dataset, relevant parameter (crop and environmental parameters) values were selected. The crop parameters were leaf number, leaf area, stem height, leaf color, and so on. The environmental parameters contained air humidity, air temperature, light intensity, soil humidity, soil temperature, soil nitrogen, phosphorus, and potassium. The simulation experiment of the lettuce growth and environmental parameters was performed.

To simplify the experiment, the data sensing experiments were performed with the objective of gathering the environmental parameters, and the crop growing area was established within 100 mm × 100 mm. Further, 200 sensing nodes and one edge computing node were randomly selected as a cluster. Each sensor node could sense 10 environmental parameters. It was assumed that the hardware and software structures of sensor nodes were the same, and the sensing time of each parameter was 3 s, according to the average value in the real application. The data size of each environmental parameter was 10 b, the effective sensing area constraint was 70% of the experiment area, and the sensing time constraint was 12 s. The details of the experimental parameters are given in Table 1.

### 5.2. Results and Analysis

**Figure 3:**
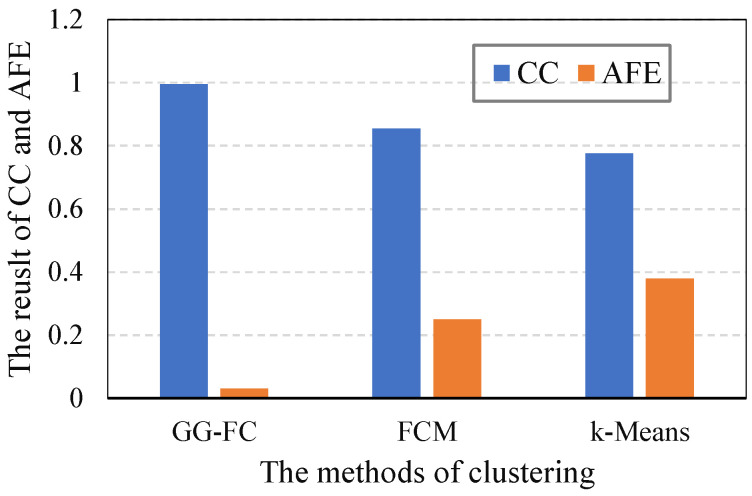
The results of CC and AFE in different methods.

**Figure 4:**
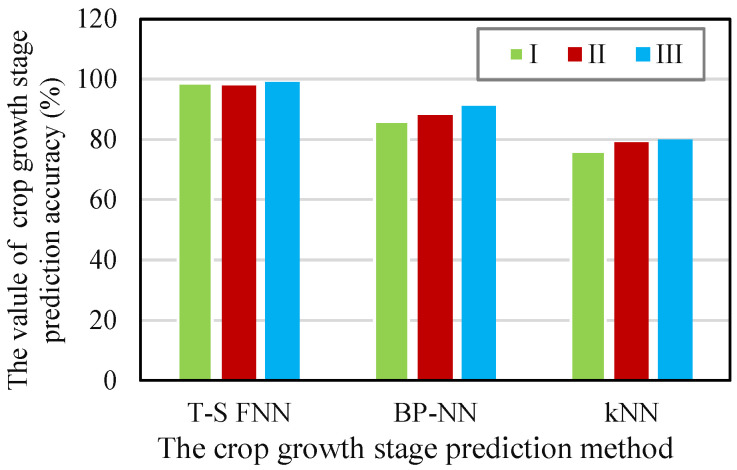
The prediction accuracy of crop growth stage in different methods.

(1) Classification coefficient (CC) and average fuzzy entropy (AFE): Based on the historical data, fuzzy clustering (FCM), *K*-means clustering (*K*-means), and GG-fuzzy clustering (GG-FC) were used to classify the growth stages. Then, the classification coefficient and average fuzzy entropy of different methods were calculated to evaluate the growth stage classification results. The results are shown in Figure 3, where it can be seen that the classification coefficient of the GG-FC was close to one, exceeding 99%; it means that the proposed method has a better division effect in crop growth stages than the others. Meanwhile, its average fuzzy entropy was less than 0.1. The results show that the proposed method had the best effect on the division of lettuce growth stages among all the methods. At the same time, the FCM classification coefficient was higher than the others; *K*-means performed the worst among all the methods. The average fuzzy entropy results were similar. Thus, the proposed GG fuzzy clustering method can be used to classify growth stages according to crop growth indicators. The proposed method can provide support for later optimization of data collection.

(2) Prediction accuracy of crop growth stage: The *K*-nearest neighbor algorithm (*K*-NN), BP neural network (BP-NN), and the proposed T-S FNN proposed were trained using the historical data collected in the experiment. Three groups of data at different growth stages were randomly selected (I(20); II(30); III(20)), the prediction verification was performed, and the prediction accuracy of the crop growth stage was calculated; the results are shown in Figure 4.

As shown in Figure 4, the prediction accuracy of the T-S FNN, BP-NN, and *K*-NN for the crop growth stage in the three test sets exceeded 96%, 80%, and 70%, respectively. The proposed T-S FNN prediction model had the highest prediction accuracy among all models, followed by the BP-NN and *K*-NN. The T-S FNN model could predict crop growth stage with high accuracy and could meet the actual requirement of more than 95% accuracy.

**Figure 5:**
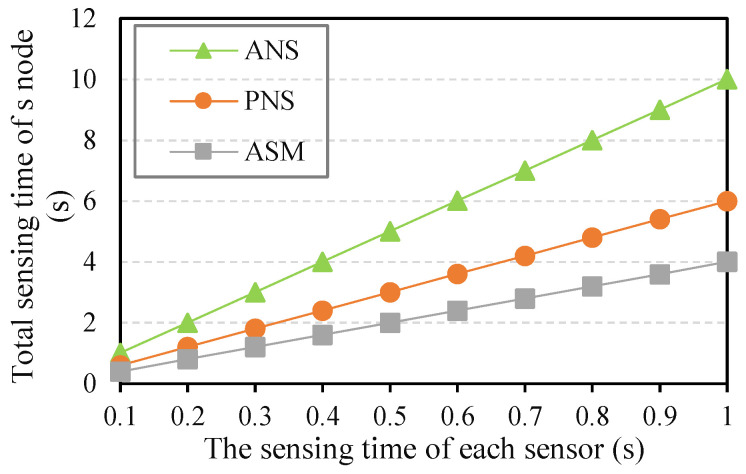
The results of sensing time in different methods.

(3) Sensing time and data collection time: As described in the related work section, there are few relevant studies on data sensing for high-value data in the entire crop lifecycle. Therefore, we divided the related sensing methods into two categories: all nodes and sensors (ANS) [28,30] and partial nodes and sensors (PFSN) [34,38]; then we chose the two methods for comparison. Under the same simulation network conditions and parameters, the experiment was performed using commonly used data sensing methods, (ANS), (PNS), and the adaptive sensing method of sensor nodes with effective area constraints (ASM). The sensing time and data collection time of different methods were obtained. The data sensing time of the three methods is shown in Figure 5, where it can be seen that as the sensing time of a single sensor increased, the corresponding sensing time of the three methods increased accordingly. The total sensing time of ASM increased significantly. The single sensor sensing time (SST) directly affected the whole sensing time of each node. However, under different SST levels, the total sensing time corresponding to the ASM method was the minimum, especially when SST was equal to 1 s; compared with ANS and PNS, the sensing time can be reduced by about 60% and 40%, respectively. The reason was that the ASM optimized the sensors involved in the data sensing work according to the crop growth stage and reduced the number of the corresponding sensors.

**Figure 6:**
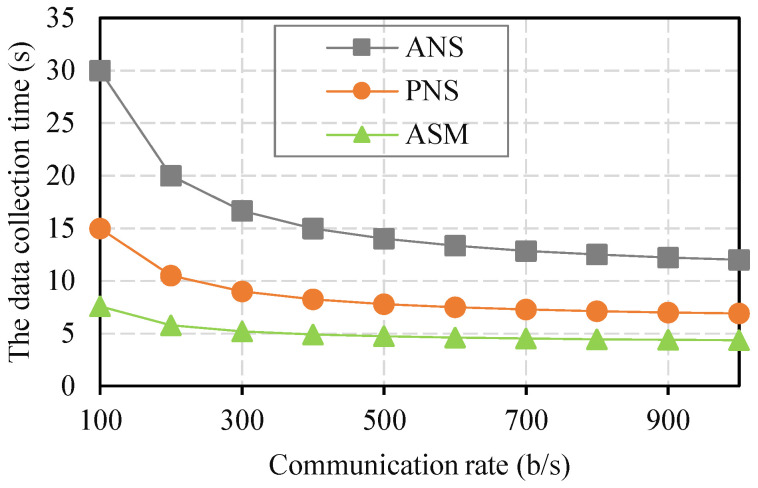
The results of data collection time in different communication rates.

The results of data collection time, including sensing time and data transmission time, for completing data collection under different communication rates in the network cluster are presented in Figure 6. As shown in Figure 6, with the continuous increase in the communication rate, the data collection time corresponding to the three schemes generally presented a downward trend. In other words, the higher the communication rate was, the shorter the data collection time was. Under the communication rate range of 100–300 b/s, the falling range (the Max collection time to the Min collection time) of the ANS was the largest, followed by those of the PNS and ASM, which was due to the small proportion of number sensing time under these conditions. Furthermore, the ASM selected nodes and sensors that were aware of the correlation analysis degree and optimized the number of nodes based on the constraint condition of the effective sensing area. Therefore, the ASM had the minimum data collection time at all communication rates. Even when the communication rate was 100 b/s, the sensing times of the ASM, PNS, and ANS were 7.6 s, 15 s, and 30 s, respectively; thus, the ASM still had minimal data collection time.

(4) Data value under different indicators: Based on the current growth stage of crops, under the constraints of sensing time, the data values of 10 sensing environmental parameters were scored by a percentage system. Then, the total value of sensing data (TDV), the data value of sensing time per unit (P-DV/ST), the data value of sensing node per unit (P-DV/NS), and the data value of effective sensing area per unit (P-DV/EA) under different indicators were calculated for the data collected in the three schemes, and the obtained results are shown in Table 2.

## 6. Conclusions

Smart agriculture requires a monitoring system to collect high-value data for the entire crop lifecycle in the context of big data at low cost. Therefore, a data collection framework was constructed by integrating sensors, edge computing, and IoT, and a data sensing strategy for the entire crop lifecycle based on edge computing was presented for improving the data value and decreasing the data collection cost. The strategy was divided into four parts, as follows: the GG fuzzy clustering crop growth stage division and key growth feature selection, current crop growth stage prediction by the T-S FNN, environmental sensing parameter optimization based on Deng’s grey correlation analysis, and adaptive sensing with effective sensing area constraint. Finally, the comparison results were obtained from an experiment. Experimental results verified that the proposed strategy can effectively divide the growth stages of the entire lifecycle, predict the current crop growth stage accurately, reduce the time of data sensing and collection, and improve the value of sensing data.

In the future, the proposed methods can be applied in practical experiments to adjust related parameters, and verify the effectiveness of the real application.

## Figures and Tables

**Table 1:** Experiment parameters.

Parameter	Value	Description
*q*	20	The number of sensor nodes
*f*	10	The sensor number of a node
t(z)	0.1–3 s	The sensing time of a sensor
$v_{c}$	10–100 b/s	The communication rate
ds(z)	10 b	The data size of a sensor
$T_{se}$	12 s	The sensing time constraint
$Ar_{\lim}$	7000 m × m	The effective sensing area

**Table 2:** The data value indicators of different methods.

Index	ANS	PNS	ASM
TDV	227.20	270.00	340.00
P-DV/ST	18.93	39.13	49.28
P-DV/NS	11.36	18.00	37.78
P-DV/EA	0.0227	0.0386	0.0447

## Data Availability

The data presented in this study are available on request from the corresponding author. The data are not publicly available due to the privacy policy of the organization.
